# Comparison of the Psychological Impact of COVID-19 on Self-Employed Private Healthcare Workers with Respect to Employed Public Healthcare Workers: Three-Wave Study during the COVID-19 Pandemic in Spain

**DOI:** 10.3390/healthcare11010134

**Published:** 2022-12-31

**Authors:** Manuel Pabón-Carrasco, Samuel Vilar-Palomo, María Luisa Gonzalez-Elena, Rocío Romero-Castillo, José Antonio Ponce-Blandon, Aurora Castro-Méndez

**Affiliations:** 1 Faculty of Nursing, Physiotherapy and Podiatry, Universidad de Sevilla, C/Avenzoar, 41009 Seville, Spain; 2 Spanish Red Cross Nursing School, Universidad de Sevilla, 41009 Sevilla, Spain

**Keywords:** COVID-19, SARS virus, health professional, psychological, pandemic, anxiety

## Abstract

(1) Background: Coronavirus disease, also called COVID-19, is a worldwide pandemic with a major impact on all aspects of the individual (health status, psychological, and economic aspects, among others). The perception of health professionals in this situation has been influenced by their economic and psychosocial situations. On the economic level, self-employed workers have no state subsidies, with the added disadvantage of not having sufficient means to cope with contagion. This could potentially have an impact on their health and indirectly on their family members, creating additional stress. The aim of this study was to determine whether there are differences in the level of anxiety of health professionals working in private practice compared to healthcare workers working in public institutions during the first three waves of COVID-19. (2) Methods: A cohort study on 517 subjects comparing anxiety between a group of health workers and a group of health professionals working in the public sector at three key moments during the pandemic was performed. (3) Results: Statistically significant differences were found between self-employed private health professionals compared to those working as public health workers. The perception of impact was worse in the self-employed; however, a higher level of anxiety was evident in public employees in all assessed domains (cognitive, physiological, and motor, *p* = 0.001). (4) Conclusions: There were significant changes when comparing the first phase between both groups; employed public healthcare workers manifested a sense of lower risk of COVID-19 contagion than privately employed professionals, who had a higher level of anxiety. In the second and third waves, negative feelings improved for both groups, and the fear of showing anxiety to the patient decreased over the course of the waves.

## 1. Introduction

Coronavirus disease, also called COVID-19, began to gain importance at the end of 2019, and on 11 March 2020, the World Health Organization (WHO) declared it a pandemic [1]. The most predominant symptoms of the disease since its appearance are the presence of a dry cough, fever, respiratory distress, fatigue, muscle aches, headache, tiredness, and diarrhea, among others, which can cause respiratory distress syndrome, septic shock, multiorgan failure, coagulation disorders, and even death [2].

In August 2022, it was estimated that around 244,632,839 people worldwide had been infected, in Europe 73,375,815, and in Spain 5,002,217 [3].

According to the latest figures, the number of deaths since the start of the pandemic is 8,7186. These figures are similar to the ones in Poland, Germany, and Ukraine, but still far from Russia, which leads the ranking with more than 172,000 deaths [4].

The presence of vaccines has been an important step against COVID-19 in the absence of an effective therapeutic treatment against the virus. In December 2020, both the European Medicines Agency (EMA) and the Food and Drug Administration (FDA) licensed the use of the Pfizer-BioNtech vaccine for the prevention of COVID-19 infection [5]. The data for this vaccine are encouraging due to the results obtained in Israel, a country where the vaccination of the population has been faster, showing a reduction of 94% of cases after the application of the second dose of the vaccine [6,7]. In turn, these agencies have approved other vaccines in recent months, such as the Moderna vaccine, with a 94.1% efficacy against COVID-19, and the AstraZeneca vaccine with a 60% efficacy [8,9].

The effects of COVID-19 are not only physical, but also psychological, both in the general population and in healthcare workers. In the general population, the diagnostic figures for anxiety and depression derived from COVID-19 are estimated to be around 20%, mainly in the population under 65 years of age [10,11]. Regarding healthcare professionals, there is an increase in the prevalence of disorders such as anxiety, stress, and depression, being more pronounced in front-line health professionals and largely generating situations of post-traumatic stress [12,13,14,15,16]. This situation of anxiety, depression, and stress can also be associated with fear, in large part due to the situation of healthcare professionals on the front line (those who deal directly with patients diagnosed with COVID-19) and the measures adopted both for their training and for their protection against the pandemic [17].

A systematic review published in July 2020, which includes 13 articles analyzing the impact of the pandemic on the mental health of nurses, doctors, and other healthcare professionals, shows a medium–high level of anxiety in these professionals, in addition to other pathologies such as depression, nervousness, and insomnia [18]. In turn, other studies also link high levels of anxiety and stress in first-line health professionals and the general population, and to a lesser extent in second-line health professionals. Regarding posttraumatic events, first- and second-line professionals have a higher prevalence compared to the general population, since the latter have less of a chance to care for people diagnosed with COVID-19 due to the isolation situation [19,20].

According to previous studies of SARS or Ebola epidemics, the onset of a sudden and life-threatening illness can lead to extreme conditions of stress and pressure in healthcare workers. Increased workload, physical exhaustion, insufficient human and material resources, risk of contagion, and nosocomial transmission have been shown to negatively affect the physical and mental well-being of professional health workers [21].

Limiting work shift hours, organizing tasks, providing rest areas, as well as broad access to and detailed rules on the use and management of protective equipment and specialized training in the treatment of patients with COVID-19 could reduce anxiety and improve the psychosocial well-being of healthcare workers [22]. Some studies have shown that the psychological impact of COVID-19 is different for healthcare workers, the general population, and patients [23,24]. A study found that nursing personnel had a higher psychological level of anxiety compared to medical personnel (professionals in public institutions). Additionally, it identified that social isolation, previous health problems, and old age were associated with higher levels of stress [25].

However, podiatrists and physiotherapists, as healthcare professionals not attached to the public health system in Spain (private professional sector), have different characteristics from the rest of the professionals. As private sector health professionals, training in the face of this pandemic, as well as protection measures in private consultations have not been similar to those in the public sphere, largely due to accessibility to resources (economic problems or material stocks), which can proportionally decrease one’s sense of well-being [26].

The impact of the pandemic on this group is different, as the level of stress and anxiety is altered not only by the physical and psychological effects derived from COVID-19, but also by the social and economic consequences that can derive from the decrease in patients who come to consultations and, therefore, the decrease in income [12].

The aim of this study was to determine whether there are differences in the level of anxiety in health professionals in the private sector compared to health professionals working at public institutions. Furthermore, it aimed to determine how anxiety has evolved linearly over the first, second, and third waves in health professionals. Through this study, the aim was to assess the most-realistic situation possible for each group and to help make decisions regarding the economic, human, and material resource measures that are necessary for each of them.

## 2. Materials and Methods

### 2.1. Study Design

A cohort study design between 27 March 2020 and 17 March 2021 was implemented. The research followed the instructions proposed by Strengthening the Reporting of Observational Studies in Epidemiology (STROBE Table S2) [27].

### 2.2. Sample

A total sample was composed of podiatrists and physiotherapists with a private work regime (self-employed work regime, where the work is carried out in a private professional office with a varying income level according to the patients who attend these private office), who participated in the study voluntarily through a questionnaire administered by professional groups on social media and through dissemination by the Professional Associations. Furthermore, the data collection for the control group was carried out in the same way to attract healthcare workers at public institutions or employed in a hospital included in the public health system, that is on a fixed payroll (doctors, nurses, technicians). Once subjects were selected, a tracking code was assigned, which allowed the subject to be anonymously identified and making a comparison between the different waves. Podiatrists and physiotherapists have close contact with patients. During the pandemic, they have had two relevant difficulties in comparison with public healthcare workers. On the one hand, they did not have the same material means to cope with COVID-19. On the other hand, these health professionals had no subsidies during the pandemic, and therefore, had to continue working in the context of limited mobility and high uncertainty [28,29,30].

In all cases, the principles established in the Declaration of Helsinki and the agreements with these principles were followed [31]. The Ethical Committee of the Colegio Oficial de Podólogos de Andalucía (COPOAN) and the Virgen de Valme Hospital, Seville (Spain), validated the ethical approval of the study (CEI; date of approval: 24 March 2020 (1114-N-20)).

A total of n = 517 participants were included in this study; 247 were active podiatrists and physiotherapists. All subjects were informed about the nature of the study and voluntarily chose to be part of it. There was a higher participation of women (68.0%), and graduates in physiotherapy (55.4%) predominated.

The average age was 38.02 years (SD 8.5; range 22–63). A control group (n = 270) of healthcare workers linked to state health institutes was recruited.

### 2.3. Procedure and Sampling Technique

The study population, active self-employed workers, was recruited through the diffusion of a questionnaire through telematic platforms and coordinated through the Professional Association. The inclusion criteria were: (1) graduates in podiatry or physiotherapy who currently work in Spain and who have their own professional activity; (2) in the same way as health professionals employed by others, both at the hospital level and outside the hospital. The exclusion criteria were: (1) podiatrists and physiotherapists working in public and private healthcare were excluded; (2) similarly, in the control group, healthcare workers working in public and private healthcare were excluded; (3) additionally, professionals with a diagnosed mental illness or in psychiatric treatment were excluded.

The information provided by the questionnaire was treated anonymously and confidentially, with limited use to achieve the objectives of this research project. The questionnaire was administered in relation to the impact produced on health personnel during the different waves. The chronology of the research coincided with the data published by the Ministry of Health, Consumption, and Social Welfare, which indicated the period of contagion in an increasing number of cases, without reaching the highest point of contagion of the COVID-19 epidemic on the date of the survey [15]. The questionnaire was identical throughout the process to measure the same variables.

**Figure 1:**
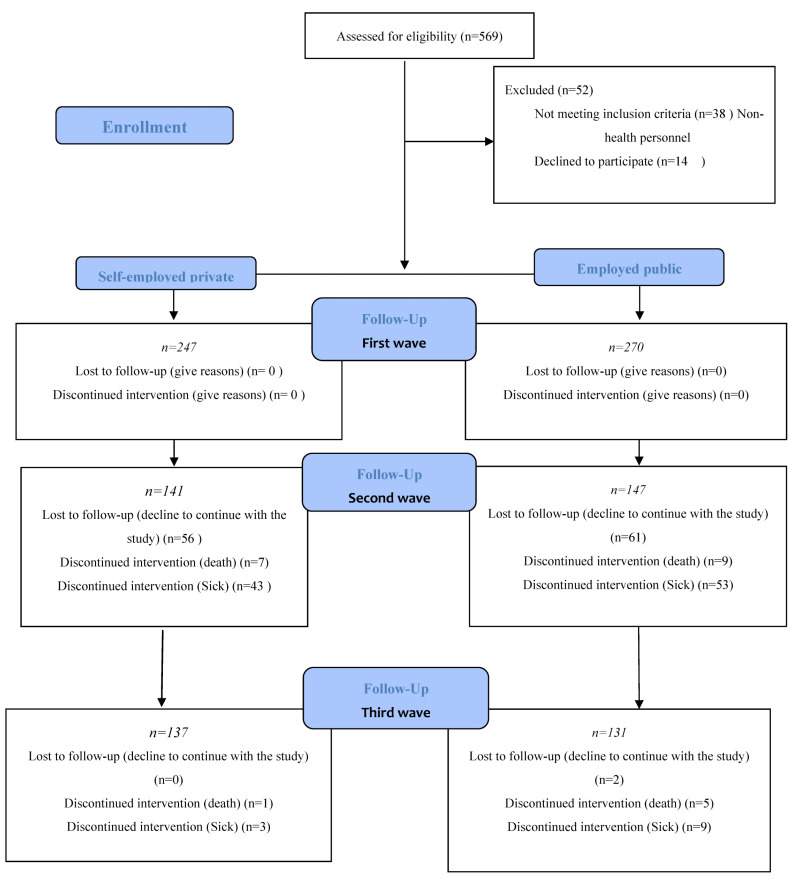
Cohort flow chart.

There were three chronological segments in the data collection: from 11 to 28 March 2020, the initial period of those infected by COVID-19, which had not reached the maximum number of affected expected in Spain; in November 2020, corresponding to the second wave of the pandemic; the third wave from December to January 2021 (Figure 1).

The sample size was calculated for a power of 0.95, with an alpha error of 0.05 and an effect size of 0.25 (G *Power 3.1.9.4, Franz Faul, University of Kiel, Kiel, Germany). This calculation yielded a necessary sample size of 258 subjects.

### 2.4. Questionnaire

Sociodemographic variables were collected using an ad hoc questionnaire relating to age, sex, relationship status, level of education (licensed, graduate, doctor), and current employment status (self-employed or salaried employee). The number of children in the household was also considered.

A previous questionnaire was adapted and used to assess perceived risk, anxiety, and behavioral responses from the general population during the first phase of the influenza A (H1N1) pandemic in the Netherlands. The scale is composed of 5 items and is categorized on a Likert scale ranging from very high, high, medium, low, and very low [32,33,34]. A readaptation was performed for the new agent, and the same structure was maintained. Additionally, new questions related to the idiosyncrasy of the group were implemented.

Furthermore, the Anxiety Situations and Response Inventory (ASRI) [35,36] was used. The ASRI is designed to assess the cognitive responses to anxiety and, in the first instance, detects the physiological and motor responses in specific situations. It identifies three independent response systems (what we think, regulated by the cognitive system; what we feel on a physical level, or the physiological system; what we do, or the behavioral motor system). The minimum score obtainable from the twelve anxiety responses after adding the scores is 0 (0 per 12 symptoms); the maximum score is 48 (4 per 12 symptoms).

Finally, the questionnaire had the following structure:

1. Sociodemographic variables: age, sex.
2. Academic education.
3. Social security system for professional activity: self-employed or salaried employee.
4. Relationship status: single, married, divorced, domestic partner, widow.
5. ASRI questionnaire; anxiety about COVID-19 [35,36].
6. Segment corresponding to the perception of the severity of COVID-19 [32,33,34].
7. Segment corresponding to the perceived effectiveness of safety measures in light of the state of emergency [32,33,34].

This questionnaire was used in a previous study related to the level of anxiety in podiatrists during the first wave [12].

An exploratory factor analysis of the questionnaires on COVID 19 and anxiety (ASRI) questions was performed. The Kaiser–Meyer–Olkin (KMO) test and the Bartlett sphericity test evaluated the applicability of factor analysis. The statistics showed an excellent adaptation of the sample (KMO 0.905; Bartlett 161,029, *p* < 0.0001), so an exploratory factor analysis of the ASRI questionnaire of anxiety faced with COVID-19 was performed. Three components with eigenvalues greater than one that met 67.45% of the variance were found. For the rotated factor matrix, the orthogonal rotation method called Varimax was used with Kaiser normalization (converted into three itineraries). In the rotations of the squared loadings, Factor 1 explained 50.51% of the variance, Factor 2, 9.59%, and Factor 3, 7.34%. Cronbach’s alpha was α = 0.85 after translation and readaptation. The ASRI questionnaire has been previously evaluated for its psychometric properties [35,36].

### 2.5. Statistical Analysis

Numerical (quantitative) variables are summarized with means and standard deviations or, in the case of highly asymmetric distributions, by medians and percentiles (P25 and P75), and frequencies and percentages were used for nonnumerical (qualitative) variables. Data were analyzed using the SPSS 24.0 computer program (SPSS Science, Chicago, IL, USA).

The Kolmogorov–Smirnov test was used to assess the distribution of the variables.

Student’s *t*-test was used to verify the score obtained by the ASRI questionnaire. On the other hand, the ANOVA test was used when the qualitative variable was polytomous. Nonparametric tests were used if the normality criteria were not met. The magnitude of the differences in pairwise comparisons was verified by the standardized effect size of Cohen’s d, Cramér’s V, and eta-squared (η2). The significance level adopted for all statistical analyzes was *p* < 0.05.

## 3. Results

### Characteristics of the Study Sample

A global sample of 517 subjects was evaluated, and statistical differences between sociodemographic variables, perception of risk, and difference between groups were assessed. (Table 1).

Significant differences were observed in both groups except for the perceived risk of being infected and relationship status. Self-employed private healthcare workers had a perception of greater impact on their health compared to healthcare workers working in the public sector. However, this situation contrasts with the information recorded in the ASRI questionnaire, where there was a greater cognitive response to anxiety and physiological and motor responses in the group of public healthcare workers.

The level of anxiety and its measurements between both groups were compared (Table S1).

During the first wave, significant differences were observed in all questions related to the ASRI questionnaire, except for concerns about the epidemic, having negative thoughts or feelings about myself, and avoidance of situations. Public employees showed greater impact in the physiological, motor, and cognitive areas.

Regarding the second wave, significant differences were observed in all questions related to the ASRI questionnaire, except for concern about the epidemic and avoidance of situations. Public employees continued to show a greater impact in the physiological, motor, and cognitive areas.

Finally, in the third wave, significant differences were observed for all the questions related to the ASRI questionnaire, except for situation avoidance. As in the previous waves, public employees continued to show a greater impact in the physiological, motor, and cognitive areas.

An intragroup analysis was carried out to assess the evolution of anxiety in its different dimensions with respect to other study variables (Table 2).

During the first wave, a high correlation with cognitive, physiological, and in some cases, motor alterations was observed in both groups. There was a relationship with gender (female), and risk perception was high in both groups.

On the other hand, in the second wave, this situation was maintained in the self-employed group (podiatrists and physiotherapists). No such relationship was observed in public health professionals. This situation was maintained in the third wave.

Only the concept that COVID-19 is detrimental to health was maintained throughout the waves.

Regarding the perception of contagion with respect to others, the relationship with psychological stress was maintained in both groups in the first two waves and disappeared in the last one.

In general terms, the cognitive area was the most affected and the motor part the least affected.

Public health workers decreased their association with stress as the waves passed. This may be due to increased knowledge and health material to prevent infection. On the other hand, self-employed workers had a problem of material resources that could not be found in previous ones. There was an increase in the cost of preventive material (masks and gloves). This money had to be assumed by the self-employed. This perpetuated mental stress in this population.

It should not be forgotten that the European funds helped to improve the conditions of public health workers.

## 4. Discussion

The aim of this study was to determine whether there were differences in the level of anxiety of health professionals working in the private (self-employed) field compared to health personnel working in public institutions (salaried employees), in addition to identifying how anxiety evolved from the first to the third wave in health professionals.

In this study, statistically significant differences were found between self-employed and salaried employees, podiatrists and physiotherapists being self-employed personnel at the private level and doctors, nurses, and auxiliary nursing care technicians employed by the public health system.

The perception of the risks of COVID-19 contagion was very high in self-employed personnel; however, the perceived anxiety was greater in salaried employees, mainly in the first wave (high risk of contagion when in contact with patients infected by COVID-19). An increase of fear felt among self-employed personnel of COVID-19 spreading can be related to the misinformation that occurred at the beginning of the first wave of the pandemic, as well as the lack of protective material. In a previous study conducted in podiatrists, anxiety was related to relationship status, where married professionals living with a family had the highest level of anxiety and fear. This could be due to the concern that they may be a possible risk of contagion to their partner and family [12], but also to the fact that a decrease in the number of patients attending their private practices means lower economic income. In other research conducted on medical and nursing health personnel, it was concluded that the most-frequent personal concern of this pandemic was related to family health and the risk of transmission to their families and patients [18,37].

The impact of the COVID-19 pandemic on the psychosocial aspect of health professionals is evident, as shown by some studies in which 40% of healthcare workers had anxiety problems [38,39]. In this study, more than 25% of public healthcare workers had perceived anxiety and approximately 20% in the case of self-employed personnel, higher in the first wave in both cases. According to an analysis of 14 studies conducted on healthcare professionals caring for patients with COVID-19, depressive symptoms were detected in 14.5% of subjects [40]. In this study, depressive symptoms were measured with the item “negative thoughts or feelings about myself”. Approximately 80% of health workers, both self-employed and salaried employees, had had negative thoughts or feelings about themselves during the first wave.

In the second and third waves, it improved only in self-employed personnel, falling to 70% and 60%, while it remained stable throughout the three waves for salaried employees. In podiatrists and physiotherapists, anxiety was higher in the first wave due to the closing of their private practices and, consequently, a sharp decrease in their income [12].

In a study conducted on healthcare professionals from two hospitals in Italy, 29.6%, 22.8%, and 44.9% of the subjects reported moderate to severe symptoms of anxiety, depression, and distress, respectively.

This study concluded that the nursing profession and female sex were associated with an increased risk of developing severe psychological symptoms during the pandemic [41]. In the case of health personnel in the hospital environment, distress and anxiety increased as the pandemic progressed and worsened in the long run [39,42].

Along the same lines, R. Rossi et al. carried out a comparison of the psychosocial impact between healthcare workers and the general population. It was found that depressive symptoms were more frequent in the general population (28.12%) and first-line health workers (28.35%) compared to second-line health workers (19.98%). Insomnia showed a prevalence of 7.82%, 6.58%, and 9.92% for the general population, second-line health workers, and first-line health workers, respectively. On the other hand, anxiety symptoms showed a prevalence of 21.25% for the general population, 18.05% for second-line health workers, and 20.55% for first-line health workers. The authors concluded that there is evidence of an impact on the mental health of healthcare workers and the general population [19].

Similarly, L. J. Labrague et al. examined the relative influence of fear of COVID-19 on nurses’ psychological distress, job satisfaction, and intention to leave the organization and profession. The authors obtained a composite COVID-19 fear scale score of 19.92. Therefore, the authors concluded that front-line nurses did not attend COVID-19-related training and those in part-time positions reported increased fears about COVID-19 [17].

On the other hand, Rathod et al. assessed the psychological impact of COVID-19; the resulting measures and future life consequences will become known over time. The results showed that 32% of participants reported suicidal thoughts. Health professionals reported mild depression and anxiety in higher proportions. Therefore, the authors concluded that there is evidence of a psychological impact on healthcare workers [20].

This statement coincides with the data obtained in this study, since perceived anxiety increased in the second and third waves.

Another parameter that was measured in this study was the fear of exhibiting anxiety towards the patient and insecurity. Fear of anxiety decreased during the course of the waves in all subjects surveyed; this may be due to increased information and the development of new protective measures, while insecurity increased slightly in both groups of workers. According to Brooks et al., insecurity and fear have increased as the pandemic has progressed due to exposure to a high level of suffering and personal exhaustion [43].

Psychological stress and perceived anxiety were reflected in symptoms such as stomach problems, perspiration, tremors, tension, and palpitations, which were more prevalent in workers of the public healthcare system than in self-employed workers, with a more pronounced effect in the first wave. Other authors also reported the psychological stress effects of the pandemic, mainly related to an increased risk of heart problems, secondary to an increase in blood pressure [44,45].

Stress can also have indirect effects such as insomnia and increased use of substances, such as tobacco [46]. In this study, the item “smoking, eating, or drinking excessively” was measured, and an increase in the first wave was observed, in addition to a higher prevalence in public health personnel, compared to self-employed personnel. Several simple methods that reduce anxiety have been identified and are part of a healthy lifestyle, such as emotional support from family and friends, a healthy and varied diet, music therapy, adequate rest, and the practice of aerobic exercise and relaxation [47].

On the other hand, it is important to emphasize that the waves did not have the same characteristics, the first wave being the phase where the greatest uncertainty was generated. During this wave, the population was confined, in addition to a new sanitary context that collapsed medical emergencies. The second wave had an important upturn after the return of summer vacations. The population continued to be infected, although with less severity. Finally, the third wave had an upturn motivated by the return of Christmas, where the population had returned to life indoors.

The study’s findings highlight the evolution that health workers have suffered throughout the pandemic with respect to their mental health. This situation is similar to other investigation conclusions, this manuscript being a pioneer in the comparison between two health groups with particular characteristics. The self-employed private sector has maintained a high level of anxiety despite not being considered first-line. They have been able to perceive their situation as difficult without receiving the media coverage of public health professionals. Health professionals have suffered significant pressure in the face of an emerging and uncontrolled threat. The lack of initial information, the fear of contagion, and the lack of material and financial resources have aggravated this situation.

As a future study, the authors propose to analyze the strategies healthcare personnel use to deal with all the physical and psychological aftereffects that remain from the COVID-19 pandemic. We believe that, in order to implement intervention programs, it is necessary to first know the current state of the population. Therefore, it was decided to carry out a study to assess the perception of anxiety in the three main waves of cases during this global pandemic.

Despite the limitations of this study, it is important to realize that it is a descriptive study that allows few inferences to be made to clinical practice. However, to reduce this limitation, three segments were considered, assessed throughout the evolution of the three waves of the pandemic. In addition, the participants were monitored to minimize bias in light of the different casuistries that can be found among the participants. Gender can also be a bias, since according to previous literature, the female sex has been linked to a greater perception of the level of anxiety. In this study, there was a majority of women, mainly in the salaried employee group, which may be influenced by nursing, an eminently female profession.

## 5. Conclusions

The results obtained showed that the evolution of the perception of healthcare workers in the different waves of the COVID-19 pandemic was remarkable.

During the first wave, public health workers expressed a greater sense of risk of COVID-19 infection than private healthcare professionals. However, both groups had a high level of anxiety and perception of risk.

In the second and third waves, negative feelings about themselves improved for salaried workers, whereas self-employed healthcare workers were unchanged in this respect.

In terms of the fear of showing anxiety in front of patients, it decreased throughout the three waves. The two groups started from different sources, which led to differences throughout the pandemic.

## Figures and Tables

**Table 1:** Sociodemographic and general data of the sample.

Category (n = 517)	Subcategories	% (n = 247)	% (n = 270)	*p* Value (ES)
		Self-Employed Private	Employed Public	
Gender ^$^	Men	32.00 (n = 79)	24.10 (n = 65)	0.029 **
	Women	68.00 (n = 168)	75.90 (n = 205)	(0.460) ^&^
Professional category ^$^	Podiatrists	44.54 (n = 110)		
	Physiotherapists	55.46 (n = 137)		
	Doctors		15.6 (n = 42)	
	Nurses		61.1 (n = 165)	0.001 ***
	Auxiliary Nurses		13.3 (n = 36)	(0.510) ^&^
	Other		10.0 (n = 27)	
Employment situation ^$^	Private Clinics	100 (n = 247)		
	Hospital		60.0 (n = 162)	
	Primary Care		32.2 (n = 87)	0.001 ***
	Nursing Homes		7.8 (n = 21)	(0.502) ^&^
Marital status ^$^	Single	27.9 (n = 69)	27.0 (n = 73)	
	Married/Domestic Partner	65.2 (n = 161)	66.7 (n = 180)	0.941
	Divorced/Widow	6.9 (n = 17)	6.3 (n = 17)	(0.214) ^&^
Children < 18 years old in the home ^$^	Yes	46.5 (n = 115)	40.4 (n = 109)	0.001 ***
	No	53.5 (n = 132)	59.6 (n = 161)	(0.415) ^&^
Risk of contracting the disease due to my age or presence of previous pathologies. ^$^	Very High	5.6 (n = 14)	0.9 (n = 2)	
	High	15.6 (n = 39)	19.7 (n = 53)	
	Medium	26.5 (n = 65)	28.5 (n = 77)	0.001 ***
	Low	22.5 (n = 55)	32.8 (n = 89)	(0.578) ^&^
	Very Low	29.8 (n = 74)	18.0 (n = 49)	
COVID-19 is harmful to my health ^$^	Very High	39.6 (n = 98)	10.1 (n = 27)	
	High	22.7 (n = 56)	28.0 (n = 76)	
	Medium	24.8 (n = 61)	38.7 (n = 104)	0.001 ***
	Low	9.8 (n = 24)	20.4 (n = 55)	(0.578) ^&^
	Very Low	3.2 (n = 8)	2.8 (n = 8)	
	Very High	18.5 (n = 46)	1.1 (n = 3)	
	High	26.0 (n = 64)	36.3 (n = 98)	
Perceived likelihood of becoming infected ^$^	Medium	35.2 (n = 87)	37.9 (n = 102)	0.001 ***
	Low	16.9 (n = 42)	22.8 (n = 62)	(0.503) ^&^
	Very Low	3.3 (n = 8)	1.8 (n = 5)	
Perceived severity ^$^	Very High	9.4 (n = 23)	11.2 (n = 30)	
	High	21.5 (n = 53)	27.3 (n = 74)	
	Medium	42.7 (n = 105)	38.9 (n = 105)	0.014 **
	Low	20.2 (n = 50)	19.2 (n = 52)	(0.512) ^&^
	Very Low	6.3 (n = 16)	3.5 (n = 9)	
	Very High	24.6 (n = 61)	0.0 (n = 0)	
Severity of COVID-19 ^$^	High	45.6 (n = 113)	28.7 (n = 77)	
	Medium	25.6 (n = 63)	33.3 (n = 90)	0.001***
	Low	3.1 (n = 8)	27.1 (n = 74)	(0.622) ^&^
	Very Low	1.0 (n = 2)	10.9 (n = 29)	
		Media (SD)	Media (SD)	*p* value
Perceived anxiety (ASRI) total first wave ^£^		19.2 (10.17)	27.31 (11.89)	0.001 *** (0.645) #
Perceived anxiety (ASRI) total second wave ^£^		16.79 (10.72)	26.02 (10.88)	0.001 *** (0.617) #
Perceived anxiety (ASRI) total third wave ^£^		18.45 (10.66)	25.83 (11.13)	0.001 *** (0.627) #
Cognitive (ASRI) first wave ^£^		8.84 (2.87)	10.50 (3.38)	0.001 *** (0.573) #
Cognitive (ASRI) second wave ^£^		8.15 (3.50)	9.91 (3.35)	0.001 *** (0.548) #
Cognitive (ASRI) third wave ^£^		8.23 (3.28)	9.71 (2.96)	0.001 *** (0.564) #
Physiological (ASRI) first wave ^£^		4.43 (4.45)	8.96 (5.52)	0.001 *** (0.594) #
Physiological (ASRI) second wave ^£^		3.70 (4.37)	8.52 (5.03)	0.001 *** (0.542) #
Physiological (ASRI) third wave ^£^		4.61 (4.67)	8.55 (5.25)	0.001 *** (0.532) #
Motor (ASRI) first wave		4.56 (3.12)	5.36 (3.28)	0.006 ** (0.518) #
Motor (ASRI) second wave		3.92 (2.94)	5.22 (2.93)	0.001 *** (0.567) #
Motor (ASRI) third wave		4.28 (2.84)	5.16 (3.26)	0.023 ** (0.509) #

Note: Chi-squared test ^$^; *t*-tests ^£^. ES: effect size; #: Cohen’s d; ^&^: Cramér’s V. Significance set at *p* < 0.05. ** *p* < 0.01; *** *p* < 0.001.

**Table 2:** Analysis of the anxiety intragroup.

First Wave	Self-Employed Private					Employed Public				
	Perceived Anxiety (ASRI) Total	ES	Cognitive (ASRI)	Physiological (ASRI)	Motor (ASRI)	Perceived Anxiety (ASRI) Total	ES	Cognitive (ASRI)	Physiological (ASRI)	Motor (ASRI)
	χ^2^ o u (*p*)		χ^2^ o u (*p*)	χ^2^ o u (*p*)	χ^2^ o u (*p*)	χ^2^ o u (*p*)		χ^2^ o u (*p*)	χ^2^ o u (*p*)	χ^2^ o u (*p*)
Gender ^†^	3175 (0.001 ***)	0.06 ^&^	3355.5 (0.003 ***)	3344 (0.002 ***)	3381.5 (0.003 ***)	5265.5 (0.011 **)	0.09 ^&^	5371 (0.018 **)	4910 (0.001 ***)	6121 (0.321)
Employment situation ^†^	3.678 (0.287)	0.04 ^#^	0.627 (0.428)	2.31 (0.128)	4.30 (0.038 *)	3.678 (0.298)	0.04 ^#^	0.610 (0.894)	8.43 (0.0.38 *)	0.72 (0.868)
Severity of COVID-19 ^†^	20.30 (0.001 ***)	0.23 ^$^	31.3 (0.001 ***)	18.6 (0.001 **)	13.01 (0.011 **)	8.56 (0.033 **)	0.17^&^	10.2 (0.017 **)	8.12 (0.043 *)	1.29 (0.729)
Risk of contracting the disease because of my age or presence of previous pathologies ^†^	21.31 (0.001 ***)	0.14 ^&^	25.77 (0.001 ***)	15.36 (0.001 ***)	14.34 (0.001 ***)	17.96 (0.001 ***)	0.16 ^&^	22.55 (0.001 ***)	11.00 (0.001 ***)	17.98 (0.001 ***)
COVID-19 is harmful for my health ^†^	16.70 (0.002 ***)	0.14 ^&^	17.81 (0.005 **)	13.58 (0.009 **)	15.56 (0.004 **)	18.70 (0.001 ***)	0.15 ^&^	21.21 (0.001 ***)	16.58 (0.001 ***)	15.06 (0.003 **)
Perceived susceptibility ^†^	56.19 (0.001 ***)	0.31 ^$^	53.96 (0.001 ***)	44.69 (0.001 ***)	37.40 (0.001 ***)	58.19 (0.001 ***)	0.32 ^$^	51.96 (0.001 ***)	47.69 (0.001 ***)	39.40 (0.001 ***)
Perceived possibility of getting infected ^†^	35.77 (0.001 ***)	0.23 ^$^	29.61 (0.001 ***)	28.02 (0.001 ***)	26.81 (0.001 ***)	37.77 (0.001 ***)	0.23 ^$^	32.11 (0.001 ***)	29.82 (0.001 ***)	28.01 (0.001 ***)
**Second Wave**	**Self-Employed Private**					**Employed Public**				
	**Perceived Anxiety (ASRI)** **Total**	**ES**	**Cognitive** **(ASRI)**	**Physiological** **(ASRI)**	**Motor** **(ASRI)**	**Perceived Anxiety (ASRI)** **Total**	**ES**	**Cognitive** **(ASRI)**	**Physiological** **(ASRI)**	**Motor** **(ASRI)**
	χ^2^ o u (*p*)		χ^2^ o u (*p*)	χ^2^ o u (*p*)	χ^2^ o u (*p*)	χ^2^ o u (*p*)		χ^2^ o u (*p*)	χ^2^ o u (*p*)	χ^2^ o u (*p*)
Gender ^†^	1597 (0.017 *)	0.07 ^&^	1610.5 (0.019*)	1687 (0.041 *)	1759.5 (0.093)	1539(0.178)	0.05 ^#^	1371(0.030)	1607(0.307)	1667(0.461)
Employment situation ^†^	0.184 (0.668)	0.03 ^#^	.420 (0.517)	0.062 (0.803)	0.066 (0.798)	8.26 (0.041 *)	0.07 ^&^	9.89 (0.019)	6.59 (0.086)	5.59(0.133)
Severity of COVID-19 ^†^	1.79 (0.617)	0.07 ^&^	4.35 (0.226)	1.59 (0.661)	9.43 (0.815)	1.67 (0.642)	0.04 ^#^	1.81 (0.611)	1.77 (0.620)	3.21 (0.360)
Risk of contracting the disease because of my age or presence of previous pathologies ^†^	24.60 (0.001 ***)	0.14 ^&^	29.50 (0.001 ***)	19.86 (0.001 ***)	15.80 (0.001 ***)	1.35 (0.903)	0.12 ^&^	0.365 (0.985)	1.04 (0.903)	3.29 (0.511)
COVID-19 is harmful for my health ^†^	18.15 (0.001 ***)	0.13 ^&^	21.67 (0.001 ***)	15.90 (0.001 ***)	13.50 (0.011 **)	18.02 (0.001 ***)	0.11 ^&^	20.47 (0.001 ***)	14.91 (0.004 **)	13.57 (0.011 **)
Perceived susceptibility ^†^	34.83 (0.001 ***)	0.27 ^$^	42.17 (0.001 ***)	28.23 (0.001 ***)	16.53 (0.002 **)	1.79 (0.774)	0.04 ^#^	1.80 (0.772)	1.65 (0.779)	0.943 (0.918)
Perceived possibility of getting infected ^†^	19.80 (0.001 ***)	0.20 ^$^	14.94 (0.005 **)	14.20 (0.007 **)	15.39 (0.004 **)	3.54 (0.471)	0.03 ^#^	0.625 (0.960)	5.16 (0.270)	4.15 (0.386)
**Third Wave**	**Self-Employed Private**					**Employed Public**				
	**Perceived Anxiety (ASRI)** **Total**	**ES**	**Cognitive** **(ASRI)**	**Physiological** **(ASRI)**	**Motor** **(ASRI)**	**Perceived Anxiety (ASRI)** **Total**	**ES**	**Cognitive** **(ASRI)**	**Physiological** **(ASRI)**	**Motor** **(ASRI)**
	χ^2^ o u (*p*)		χ^2^ o u (*p*)	χ^2^ o u (*p*)	χ^2^ o u (*p*)	χ^2^ o u (*p*)		χ^2^ o u (*p*)	χ^2^ o u (*p*)	χ^2^ o u (*p*)
Gender ^†^	1294.5 (0.001 ***)	0.06 ^&^	1330.5 (0.001 ***)	1271 (0.001 ***)	1638.5 (0.059)	1260(0.101)	0.03 ^#^	1385 (0.330)	1230 (0.071)	1233 (0.073)
Employment situation ^†^	1.283 (0.257)	0.05 ^#^	0.707 (0.400)	0.504 (0.478)	3.39 (0.066)	0.477 (0.924)	0.04 ^#^	2.00 (0.572)	0.637 (0.888)	0.201 (0.977)
Severity of COVID-19 ^†^	19.98 (0.001 ***)	0.21 ^$^	26.47 (0.001 ***)	14.44 (0.006 **)	13.60 (0.009 **)	3.49 (0.479)	0.14 ^&^	5.24(0.263)	2.39 (0.664)	4.88 (0.299)
Risk of contracting the disease because of my age or presence of previous pathologies ^†^	17.53 (0.001 ***)	0.14 ^&^	20.92 (0.001 ***)	9.43 (0.050 *)	19.18 (0.001 ***)	3.07 (0.546)	0.12 ^&^	4.98 (0.288)	3.78 (0.436)	2.54 (0.637)
COVID-19 is harmful for my health ^†^	19.59 (0.001 ***)	0.17 ^&^	28.67 (0.001 ***)	13.73 (0.008 **)	10.60 (0.031 **)	19.04 (0.001 ***)	0.16 ^&^	26.17 (0.001 ***)	12.61 (0.008 **)	9.62 (0.031 **)
Perceived susceptibility ^†^	47.01 (0.001 ***)	0.30 ^$^	59.52 (0.001 ***)	35.91 (0.001 ***)	22.71 (0.001 ***)	3.52 (0.475)	0.04 ^#^	2.64 (0.620)	3.63 (0.458)	4.62 (0.329)
Perceived possibility of getting infected ^†^	4.30 (0.366)	0.05 ^#^	6.37 (0.173)	3.02 (0.553)	7.81 (0.099)	1.36 (0.850)	0.05 ^#^	0.731 (0.947)	1.75 (0.781)	2.65 (0.617)

Note: ^†^ Wilcoxon test and Cohen’s d; ES: effect size; #: small ES; ^&^: medium ES; ^$^: large ES. Significance set at *p* < 0.05. * *p* < 0.05; ** *p* < 0.01; *** *p* < 0.001.

## Data Availability

The data that support the findings of this study are available upon request from the corresponding author. The data are not publicly available due to privacy or ethical restrictions.

## Supplementary Materials

The following Supporting Information can be downloaded at: https://www.mdpi.com/article/10.3390/healthcare11010134/s1. Table S1: Valuation of the level of anxiety; Table S2: STROBE Statement—checklist of items that should be included in reports of observational studies.
