# Supplementary material for: Comparison of the Psychological Impact of COVID-19 on Self-Employed Private Healthcare Workers with Respect to Employed Public Healthcare Workers: Three-Wave Study during the COVID-19 Pandemic in Spain
Source: Healthcare (Basel). 2022 Dec 31;11(1):134. doi: 10.3390/healthcare11010134 (PMC9819057; doi:10.3390/healthcare11010134)
Supplement: Supplementary file 1 [file healthcare-11-00134-s001.zip › healthcare-1976670-supplementary.pdf]

healthcare

MDPI

Table S1. Valuation of the level of anxiety.

**First wave$**	**Self-employed private n=247**					**Employed public n=270**					
Perceived anxiety (ASRI)	Almost always	Often	At times	Not often	Almost never	Almost always	Often	At times	Not often	Almost never	*p* value
1. Concerned about the COVID-19	52.0(n=129)	32.7(n=81)	11.9(n=29)	3.5(n=8)	0.0(n=0)	51.1(n=138)	43.3(n=117)	5.6(n=15)	0.0(n=0)	0.0(n=0)	.389
2. Negative thoughts or feelings about myself	14.9(n=37)	29.2(n=72)	34.7(n=86)	13.9(n=34)	7.4(n=18)	16.7(n=45)	31.5(n=85)	33.0(n=89)	15.2(n=41)	3.7(n=10)	.300
3. Fear of my anxiety being noticed and what people will think if that happens	10.4(n=26)	17.8(n=44)	23.8(n=59)	15.3(n=38)	32.7(n=81)	18.1(n=49)	27.4(n=74)	21.1(n=57)	16.7(n=45)	16.7(n=45)	.001***
4. Stomach problems	4.0(n=10)	10.4(n=26)	20.3(n=50)	12.9(n=32)	52.5(n=130)	17.7(n=37)	27.0(n=73)	21.1(n=57)	11.5(n=31)	26.7(n=72)	.001***
5. Perspiration	0.5(n=1)	5.9(n=15)	12.9(n=32)	12.9(n=32)	67.8(n=167)	12.2(n=33)	18.1(n=49)	21.5(n=58)	14.4(n=39)	33.7(n=91)	.001***
6. Tremors	0.0(n=0)	2.0(n=5)	8.4(n=21)	11.9(n=29)	77.7(n=192)	6.3(n=17)	8.9(n=24)	18.1(n=49)	16.3(n=44)	50.4(n=136)	.001***
7. Tension	8.4(n=21)	16.8(n=42)	20.3(n=50)	13.9(n=34)	40.6(n=100)	24.4(n=66)	34.4(n=93)	18.5(n=50)	10.7(n=29)	11.9(n=32)	.001***
8. Palpitations, rapid heartbeat	6.9(n=17)	10.4(n=26)	17.8(n=44)	15.8(n=39)	49.0(n=121)	16.3(n=44)	23.0(n=62)	20.7(n=56)	16.7(n=45)	23.3(n=63)	.001***
9. Repetitive movements (feet, hands, scratching)	5.0(n=12)	14.4(n=36)	13.9(n=34)	12.9(n=32)	54.0(n=133)	10.7(n=29)	23.3(n=63)	19.6(n=53)	15.9(n=43)	30.4(n=82)	.001***
10. Smoking, eating, or drinking excessively	12.9(n=32)	12.4(n=37)	19.3(n=48)	19.8(n=49)	35.6(n=88)	19.3(n=52)	20.7(n=56)	17.0(n=46)	17.4(n=47)	25.6(n=59)	.001***
11. Avoiding situations	18.3(n=45)	24.8(n=61)	20.8(n=51)	16.8(n=41)	19.3(n=48)	10.7(n=29)	21.9(n=59)	23.3(n=63)	21.9(n=59)	22.2(n=60)	.022**

12. Insecurity	0.0(n=0)	26.7(n=66)	28.2(n=70)	25.7(n=63)	19.3(n=48)	27.8(n=75)	26.7(n=72)	22.6(n=61)	12.6(n=34)	10.4(n=28)	.001***

**Second Wave£**	Self-employed private n=141					Employed public n=147					
	Almost always	Often	At times	Not often	Almost never	Almost always	Often	At times	Not often	Almost never	p value
1. Concerned about the COVID-19	33.3(n=47)	42.6(n=60)	20.6(n=29)	3.5(n=5)	0.0(n=0)	36.2(n=53)	51.8(n=76)	10.6(n=16)	1.4(n=2)	0.0(n=0)	.106
2. Negative thoughts and feelings about myself	8.5(n=12)	25.5(n=36)	38.3(n=54)	18.4(n=26)	9.2(n=13)	14.2(n=21)	30.5(n=45)	36.9(n=54)	14.2(n=21)	4.3(n=6)	.020**
3. Fear of my anxiety being noticed and what people will think if that happens	7.1(n=10)	11.3(n=16)	27.7(n=39)	31.0(n=22)	31.9(n=45)	12.8(n=19)	29.8(n=44)	19.9(n=29)	19.9(n=29)	17.7(n=26)	.001***
4. Stomach problems	2.1(n=3)	8.5(n=12)	17.0(n=24)	19.1(n=27)	53.2(n=75)	5.7(n=8)	31.2(n=46)	27.0(n=40)	11.3(n=16)	24.8(n=37)	.001***
5. Perspiration	2.1(n=3)	2.8(n=4)	10.6(n=15)	14.2(n=20)	70.2(n=99)	5.0(n=7)	24.1(n=35)	24.1(n=35)	15.6(n=24)	31.2(n=46)	.001***
6. Tremors	1.4(n=2)	2.8(n=4)	5.0(n=7)	14.2(n=20)	76.6(n=108)	2.8(n=4)	12.1(n=18)	16.3(n=24)	16.3(n=24)	52.5(n=77)	.001***
7. Tension	2.8(n=4)	11.3(n=16)	19.9(n=28)	17.0(n=24)	48.9(n=69)	18.4(n=27)	36.9(n=56)	19.9(n=29)	12.1(n=17)	12.8(n=18)	.001***
8. Palpitations, rapid heartbeat	2.8(n=4)	10.6(n=15)	13.5(n=19)	19.1(n=27)	53.9(n=76)	12.8(n=18)	23.4(n=34)	23.4(n=34)	14.2(n=21)	26.2(n=39)	.001***
9. Repetitive movements (feet, hands, scratching)	3.5(n=5)	8.5(n=12)	16.3(n=23)	13.5(n=19)	58.2(n=82)	7.1(n=10)	24.1(n=36)	25.5(n=37)	11.3(n=17)	31.9(n=47)	.001***
10. Smoking, eating, or drinking excessively	5.0(n=7)	12.8(n=18)	29.1(n=41)	22.7(n=32)	30.5(n=43)	18.4(n=27)	22.7(n=34)	17.0(n=25)	19.9(n=29)	22.0(n=32)	.001***
11. Avoiding situations	10.6(n=15)	29.6(n=29)	21.3(n=30)	20.6(n=29)	38.0(n=27)	7.1(n=10)	21.3(n=32)	24.1(n=35)	22.7(n=33)	24.8(n=37)	.849

12. Insecurity	9.2(n=13)	17.0(n=24)	27.0(n=38)	22.0(n=31)	24.8(n=35)	19.9(n=30)	32.6(n=48)	19.9(n=29)	15.6(n=23)	12.1(n=17)	.001***

**Third Wave α**	Self-employed private n=137					Employed public n=131					
	Almost always	Often	At times	Not often	Almost never	Almost always	Often	At times	Not often	Almost never	*p* value
1. Concerned about the COVID-19	52.0(n=105)	46.0(n=63)	13.1(n=18)	2.9(n=4)	0.0(n=0)	27.3(n=36)	47.0(n=62)	25.0(n=33)	0.8(n=1)	0.0(n=0)	.028**
2. Negative thoughts or feelings about myself	8.0(n=11)	25.5(n=35)	34.3(n=47)	24.8(n=34)	7.3(n=10)	8.3(n=11)	35.6(n=47)	35.6(n=47)	15.9(n=21)	4.5(n=6)	.037**
3. Fear of my anxiety being noticed and what people will think if that happens	5.1(n=7)	13.9(n=19)	26.3(n=36)	22.6(n=31)	32.1(n=44)	10.6(n=14)	31.1(n=41)	27.3(n=36)	15.9(n=21)	15.2(n=20)	.001***
4. Stomach problems	5.8(n=8)	14.6(n=20)	12.4(n=17)	21.2(n=29)	46.0(n=63)	11.4(n=15)	23.5(n=31)	23.5(n=31)	13.6(n=18)	28.0(n=37)	.001***
5. Perspiration	2.9(n=4)	12.4(n=17)	10.2(n=14)	13.9(n=19)	60.6(n=83)	6.8(n=9)	19.7(n=26)	22.7(n=30)	14.4(n=19)	36.4(n=48)	.001***
6. Tremors	0.7(n=1)	0.7(n=1)	11.0(n=8)	13.9(n=19)	76.6(n=105)	3.8(n=5)	6.8(n=9)	23.5(n=31)	18.2(n=24)	47.7(n=63)	.001***
7. Tension	7.3(n=10)	10.2(n=14)	24.8(n=34)	21.9(n=30)	35.8(n=49)	18.9(n=25)	35.6(n=47)	23.5(n=31)	11.4(n=15)	10.6(n=14)	.001***
8. Palpitations, rapid heartbeat	3.6(n=5)	12.4(n=17)	15.3(n=21)	16.1(n=22)	52.6(n=72)	12.9(n=17)	25.0(n=33)	22.0(n=29)	19.7(n=26)	20.5(n=27)	.001***
9. Repetitive movements (feet, hands, scratching)	5.8(n=8)	8.8(n=12)	14.6(n=20)	16.8(n=23)	54.0(n=74)	8.3(n=11)	22.0(n=29)	20.5(n=27)	21.2(n=28)	28.0(n=37)	.001***
10. Smoking, eating or drinking excessively	5.1(n=7)	19.0(n=26)	13.9(n=19)	24.1(n=33)	38.0(n=52)	12.9(n=17)	19.7(n=26)	22.0(n=29)	16.7(n=22)	28.8(n=38)	.015**
11. Avoiding situations	12.4(n=17)	24.8(n=34)	28.5(n=39)	22.6(n=31)	11.7(n=16)	11.4(n=15)	22.0(n=29)	24.2(n=32)	23.5(n=31)	18.9(n=25)	.186

healthcare MDPI

12. Insecurity	5.8(n=8)	19.7(n=27)	29.2(n=40)	24.1(n=33)	21.2(n=29)	18.9(n=25)	29.5(n=39)	30.3(n=40)	12.1(n=16)	9.1(n=12)	.001***

Note: Chi-square test is applied. Significance set at $p < 0.05$. * p <0,05; ** p <0,01; *** p <0,001. From March to June of 2020$; From September to November of 2020 £ ;From January to March of 2021 α.

healthcare

Table S2: STROBE Statement—checklist of items that should be included in reports of observational studies

	Item No	Recommendation	Page No
**Title and abstract**	1	(*a*) Indicate the study's design with a commonly used term in the title or the abstract	1
		(*b*) Provide in the abstract an informative and balanced summary of what was done and what was found	
**Introduction**			
Background/rationale	2	Explain the scientific background and rationale for the investigation being reported	1-2
Objectives	3	State specific objectives, including any prespecified hypotheses	2
**Methods**			
Study design	4	Present key elements of study design early in the paper	3
Setting	5	Describe the setting, locations, and relevant dates, including periods of recruitment, exposure, follow-up, and data collection	3
Participants	6	(*a*) *Cohort study*—Give the eligibility criteria, and the sources and methods of selection of participants. Describe methods of follow-up<br>*Case-control study*—Give the eligibility criteria, and the sources and methods of case ascertainment and control selection. Give the rationale for the choice of cases and controls<br>*Cross-sectional study*—Give the eligibility criteria, and the sources and methods of selection of participants	3
		(*b*) *Cohort study*—For matched studies, give matching criteria and number of exposed and unexposed<br>*Case-control study*—For matched studies, give matching criteria and the number of controls per case	
Variables	7	Clearly define all outcomes, exposures, predictors, potential confounders, and effect modifiers. Give diagnostic criteria, if applicable	4

Data sources/ measurement	8*	For each variable of interest, give sources of data and details of methods of assessment (measurement). Describe comparability of assessment methods if there is more than one group	*4*
Bias	9	Describe any efforts to address potential sources of bias	
Study size	10	Explain how the study size was arrived at	
Quantitative variables	11	Explain how quantitative variables were handled in the analyses. If applicable, describe which groupings were chosen and why	
Statistical methods	12	(*a*) Describe all statistical methods, including those used to control for confounding	3
		(*b*) Describe any methods used to examine subgroups and interactions	
		(*c*) Explain how missing data were addressed	
		(*d*) *Cohort study*—If applicable, explain how loss to follow-up was addressed<br>*Case-control study*—If applicable, explain how matching of cases and controls was addressed<br>*Cross-sectional study*—If applicable, describe analytical methods taking account of sampling strategy	3
		(*e*) Describe any sensitivity analyses	4

Results

Participants	13*	(a) Report numbers of individuals at each stage of study—eg numbers potentially eligible, examined for eligibility, confirmed eligible, included in the study, completing follow-up, and analysed	3
		(b) Give reasons for non-participation at each stage	3
		(c) Consider use of a flow diagram	3
Descriptive data	14*	(a) Give characteristics of study participants (eg demographic, clinical, social) and information on exposures and potential confounders	5
		(b) Indicate number of participants with missing data for each variable of interest	
		(c) *Cohort study*—Summarise follow-up time (eg, average and total amount)	3

healthcare MDPI

Outcome data	15*	*Cohort study*—Report numbers of outcome events or summary measures over time	*5-6*
		Case-control study—Report numbers in each exposure category, or summary measures of exposure	
		Cross-sectional study—Report numbers of outcome events or summary measures	
Main results	16	(*a*) Give unadjusted estimates and, if applicable, confounder-adjusted estimates and their precision (eg, 95% confidence interval). Make clear which confounders were adjusted for and why they were included	5-10
		(*b*) Report category boundaries when continuous variables were categorized	
		(*c*) If relevant, consider translating estimates of relative risk into absolute risk for a meaningful time period	
Other analyses	17	Report other analyses done—eg analyses of subgroups and interactions, and sensitivity analyses	
**Discussion**			
Key results	18	Summarise key results with reference to study objectives	11
Limitations	19	Discuss limitations of the study, taking into account sources of potential bias or imprecision. Discuss both direction and magnitude of any potential bias	12
Interpretation	20	Give a cautious overall interpretation of results considering objectives, limitations, multiplicity of analyses, results from similar studies, and other relevant evidence	11
Generalisability	21	Discuss the generalisability (external validity) of the study results	11-12
**Other information**			
Funding	22	Give the source of funding and the role of the funders for the present study and, if applicable, for the original study on which the present article is based	12

*Give information separately for cases and controls in case-control studies and, if applicable, for exposed and unexposed groups in cohort and cross-sectional studies.
